# Video summarization using line segments, angles and conic parts

**DOI:** 10.1371/journal.pone.0181636

**Published:** 2017-11-09

**Authors:** Md Musfequs Salehin, Manoranjan Paul, Muhammad Ashad Kabir

**Affiliations:** School of Computing and Mathematics, Charles Sturt University, Bathurst, NSW-2795, Australia; Kaohsiung Medical University, TAIWAN

## Abstract

Video summarization is a process to extract objects and their activities from a video and represent them in a condensed form. Existing methods for video summarization fail to detect moving (dynamic) objects in the low color contrast area of a video frame due to the pixel intensities of objects and non-objects are almost similar. However, edges of objects are prominent in the low contrast regions. Moreover, to represent objects, geometric primitives (such as lines, arcs) are distinguishable and high level shape descriptors than edges. In this paper, a novel method is proposed for video summarization using geometric primitives such as conic parts, line segments and angles. Using these features, objects are extracted from each video frame. A cost function is applied to measure the dissimilarity of locations of geometric primitives to detect the movement of objects between consecutive frames. The total distance of object movement is calculated and each video frame is assigned a probability score. Finally, a set of key frames is selected based on the probability scores as per user provided skimming ratio or system default skimming ratio. The proposed approach is evaluated using three benchmark datasets—BL-7F, Office, and Lobby. The experimental results show that our approach outperforms the state-of-the-art method in terms of accuracy.

## Introduction

Due to the advancement of technology, video surveillance has been used widely in emerging places to help ensure a safe and secure life style. Government, public safety organizations, and transportation agencies mainly rely on real-time video surveillance systems for security, traffic management, and emergency operations. Surveillance video cameras are setup in offices, railway stations, bus stops and other places. These cameras are used to monitor the activities within these places by trained professionals who always observe video from the monitoring centre. To investigate a crime or to find any specific events from a long video recording, it can take many work hours [1]. Furthermore, to store long videos require a huge memory space [1]. Therefore, it is essential to develop a method for extracting the most informative video frames from the long consecutive video stream.

Video summarization (VS) is a process to extract the most informative set of frames known as key frames or a set of video fragments from the original video. The main purpose of VS is to generate a short video so that an observer can get a complete idea about all the high priority entities and events. VS must be as concise as possible and should contain all significant contents of the entire video. It should also maintain continuation of information and be free of repetition without losing any important video data. To summarize a video stream by considering all these properties, it is necessary to extract some important features of the video. These features are later applied to construct a very short version of the original video. Objects and people within a video play an important role for video summarization [1]. The reason is that events in a video are usually represented by objects/people and their activities [2]. Moreover, objects/people in a video carry the high-level semantic information [3]. In addition to this, human beings usually pay more attention to moving (dynamic) objects in a video [4] [5]. Therefore, objects/people and their activities in a video are mainly extracted for generating video summarization.

It remains a challenging problem to extract dynamic objects from a video with low contrast, illumination change, noise, and multimodal dynamic environment [6] [7]. However, edges of objects are prominent in the low contrast regions [6] and less sensitive to illumination change and the multimodal environment [8]. Moreover, the problems of the methods based on edge pixels are sensitive to variation of shape and position [8]. To overcome these problems, edge-segments (groups of connected sequential edge pixels) can be applied. However, edge-segments based methods are not robust to local shape distortion and shape matching [9] [10]. To represent objects, geometric primitives (such as lines, arcs) are higher level and more distinguishable descriptors than edge-pixels or edge-segments [9] [10]. These primitives have some special properties. They are independent of object size, efficient for comparisons and matching, and invariant to scale and viewpoint changes. Therefore, these geometric primitives have the capability to represent objects with complex shapes and structures effectively. Furthermore, they often play a major role in the human cognitive system due to their discriminative power [11].

The existing methods for object detection apply complete circles or ellipses to represent curve fragments [10]. However, sometimes complete circles or ellipses may not be found for part of an object due to the actual shape of the part, low color contrast, illumination changes, or camera motion. Moreover, in the real world, part of an object can be a circular, elliptical, parabolic, or hyperbolic curve. As a result, the object detection methods proposed with the circular arc do not fit accurately with an elliptical, or a parabolic or a hyperbolic curve. On the other hand, the elliptical arc does not fit perfectly with a parabolic or a hyperbolic curve. However, a conic part can easily be fitted for any type of curves (circular, elliptical, parabolic or hyperbolic).

In this paper, a novel approach is introduced for extracting dynamic objects applying geometric primitives such as line segments, angles and conic parts, and for generating a summary of a long video. The straight contours, corners, and curved contours of an object are presented by line segments, angles, and conic (circle, ellipse, parabola, and hyperbola) parts respectively. For this purpose, an edge image is generated from a video frame by applying the Canny edge detection method. After that, lists of connected edge-segments and line segments fitted with each edge-segment are obtained from the edge image by applying the method developed by Kovesi [12]. A single line segment is considered as a straight line. Line segments with two lines are modelled as angles. Edge segments and line segments with more than two lines are matched with conic parts using Pascal’s Theorem [13] [14]. After constructing geometric primitives, their displacements between two consecutive frames are calculated by applying a new method for measuring the positional dissimilarity to include the activities of objects within a video. A probability score is assigned to each frame based on the displacements of geometric primitives. The frames are sorted based on their higher probability score. Finally, a set of key frames is selected from this sorted list based on the default skimming ratio or user preferred skimming ratio.

There are several advantages of using Pascal’s theorem for detecting curve segments. This method does not require calculating the center, major or minor axes for detecting conic shape objects. Furthermore, a one-step process can detect any type of conics (circle, ellipse, parabola, and hyperbola) or conic parts. Moreover, it does not require any parameter for conic part construction. To construct conic, Hough Transform (HT) requires higher dimensional parameter space. For example, an ellipse can be defined by five parameters, such as its center, the major axis, the minor axis and the orientation. Therefore, *O*(*N*^5^) space is required for an ellipse to accumulate the parameter space, where *N* is the size of each dimension of the parameter space. Moreover, finding an optimal threshold for selecting an ellipse from high dimensional space is another problem [15]. A large threshold may have a poor influence on accurate ellipse detection while a small threshold may lead to missing the true ellipses. Furthermore, conic detection methods based on the algebraic equation present some problems. The main disadvantages are that this method is numerically unstable [16] and it does not have any geometric interpretation [17]. To overcome these problems, a conic part is constructed based on Pascal’s theorem in the proposed method. To construct a conic part using Pascal’s theorem, two tangents at two end-points of a curve are necessary. The existing methods for tangent estimation construct a tangent on a digital curve based on a parameter [18] [19]. However, finding an optimal parameter value is one of the main problems of these methods. To solve this problem, a new parameter-free tangent estimation method based on Pascal’s theorem is proposed. The advantage of applying Pascal’s theorem is that it does not require any parameters to construct tangents on the unsmoothed digital curves.

The key contributions of the proposed method for video summarization are as follows:

1. A new parameter-less tangent estimation method is proposed for conic part construction;
2. Conic parts are applied to model curve contour for object detection instead of circular or elliptical arcs;
3. A new method for dissimilarity measure of geometric primitives is proposed for recognizing the activity of objects;
4. Geometric primitives, such as line segments, angles and conic parts are applied for extracting objects in a video with low contrast or illumination changes.

The remaining of this paper is organized as follows. Section II describes the related work proposed in the literature on video summarization methods in recent years. A brief description of Pascal’s theorem is provided in Section III. The detail of the proposed method is discussed in Section IV. Extensive experimental results as well as an analytical discussion are provided in Section V and concluding remarks are presented in Section VI.

## Related work

Objects/people play the most significant role in a video for summarization. In [20], a set of similar objects is trained to build a model and similar objects are extracted using this model for summarization. A part-based object movement framework is proposed in [21] for video synopsis generation. Object bank and object-like windows are applied to extract objects and are then utilized to detect objects for story-driven egocentric video summarization in [22]. A complementary background model is proposed in [23] to extract moving objects and video summarization. Pixel-based motion energy and edge features are combined in [24] to detect object and to summarize video. A background subtraction method is applied in [25] to detect foreground objects for video summarization. Eye tracking data is applied in [26] for important object detection from a video. In [27], important objects from a video are detected using features and object segmentation. Aggregated Channel Features (ACF) detection and a background subtraction technique are applied for object detection in [28] for surveillance video synopsis generation. The non-parametric background model is employed to extract moving objects in [29] for producing a condensed version of a surveillance video. A background subtraction method is also used in [30] for object detection. In [2], robust motion and cluster analysis are utilized for object location detection for summarizing rushes video. For generating storyboard, important objects are detected by a min-cut method in [31].

In addition, a Bayesian foraging strategy is applied in [32] for objects and their activities detection to summarize a video. The grid background model is applied in [33] for object detection. In [34], a key-point matching based video segmentation method is employed to locate the visual objects in a video. Spatio-temporal slices are applied in [5] to select the states of the object motion for video summarization. J Value Segmentation (JSEG) algorithm is applied in [35] for object detection to extract key frames from a wildlife video. Latent Dirichlet Allocation (LDA) is applied in [36] for detecting objects and their activities for video summarization. The background subtraction method is applied in [37] for human objects detection. Objects in a video are described by Histograms of Optical Flow Orientations (HOFO) in [38] and their activities are detected by the Support Vector Machine (SVM) classifier. In [39], moving object and motion information calculated in spatial and frequency domain are combined for video summarization.

Moreover, image signature is applied for foreground object detection and then fused with motion information to summarize egocentric video in [40]. A modularity cut algorithm is employed in [41] to track objects and use this information for summary generation. Faces of human objects are applied for movie summarization in [42]. Moving objects are detected in [43] using the forward/backward frame differencing method. Foreground object and saliency map difference are applied in [44] for surveillance video summarization.

Recently, a surveillance video summarization method is proposed in [1]. Single view summarization is generated in the approach for each sensor independently. For this purpose, MPEG-7 color layout descriptor is applied to each video frame and an online-Gaussian mixture model (GMM) is used for clustering. The key frames are selected based on the parameters of cluster. As the decision of selecting or neglecting a frame is performed based on the continuous updates of these clustering parameters, a video segment is extracted instead of key frames. The video summarization technique using a single type descriptor (i.e., color descriptor) in frame-level with on-line learning (i.e., GMM) strategy provides very good performance if the video has uni-modal phenomenon, however, the technique may not perform well if the video has multi-modal phenomena such as illumination change, variation of local motion, or occlusion.

To the best of our knowledge, existing approaches for video summarization did not apply geometric primitives (line segments, angles, and conic parts) although they have the capabilities to represent objects with complex shapes and structures effectively in challenging environments such as video with low contrast and illumination change. These geometric primitives have several important properties as mentioned in the introduction section. For example, they are independent of object size, efficient for comparisons and matching, and invariant to scale and viewpoint changes. Thus, in this paper a new video summarization method utilizing geometric primitives is proposed.

## Pascal’s theorem

**Fig 1:**
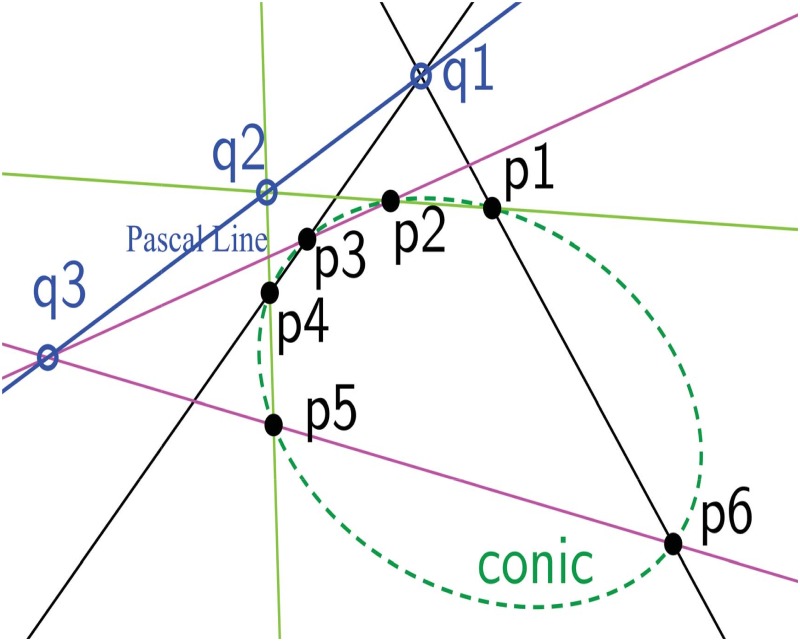
Explanation of Pascal’s theorem. A hexagon (*p*1*p*2*p*3*p*4*p*5*p*6) is inscribed in a conic represented by green dotted curve. The three pairs of opposite vertices of the hexagon intersects at *q*1, *q*2, and *q*3. The line connecting *q*1, *q*2, and *q*3 is Pascal line.

In this work, the curve segments (conic parts) are extracted using Pascal’s theorem [13] [14]. Therefore, a brief introduction about Pascal’s theorem is provided in this section. Pascal’s theorem states that when a hexagon (no three points are co-linear and no parallel lines) is inscribed in a conic, the three pairs of opposite sides meet three points of intersection. These three points are collinear. This line is called Pascal line [13] [14]. In Fig 1, *p*1, *p*2, *p*3, *p*4, *p*5, and *p*6 are six vertices of a hexagon inscribed in a conic (green dotted ellipse) where no three vertices are co-linear and sides of the hexagon are not parallel. The pair of opposite sides is represented with the same color. The point *q*1 is the intersection between opposite sides *p*6*p*1 (black line) and *p*4*p*3 (black line). The intersection of opposite sides *p*1*p*2 (light green line) and *p*5*p*4 (light green line) is *q*2. The opposite sides *p*2*p*3 (magenta line) and *p*6*p*5 (magenta line) meet at *q*3 point. According to Pascal’s theorem, the intersecting points (*q*1, *q*2 and *q*3) are co-linear and the line connecting these points is Pascal line (blue line).

Pascal’s theorem can also be applied when five vertices from a hexagon are provided. The sixth vertex can be calculated using the provided five vertices. This sixth vertex will also be on the conic sections and satisfy the property of co-linearity. Interested readers are referred to [45] for more details regarding conic construction using five points by Pascal’s theorem.

## The proposed approach

**Fig 2:**
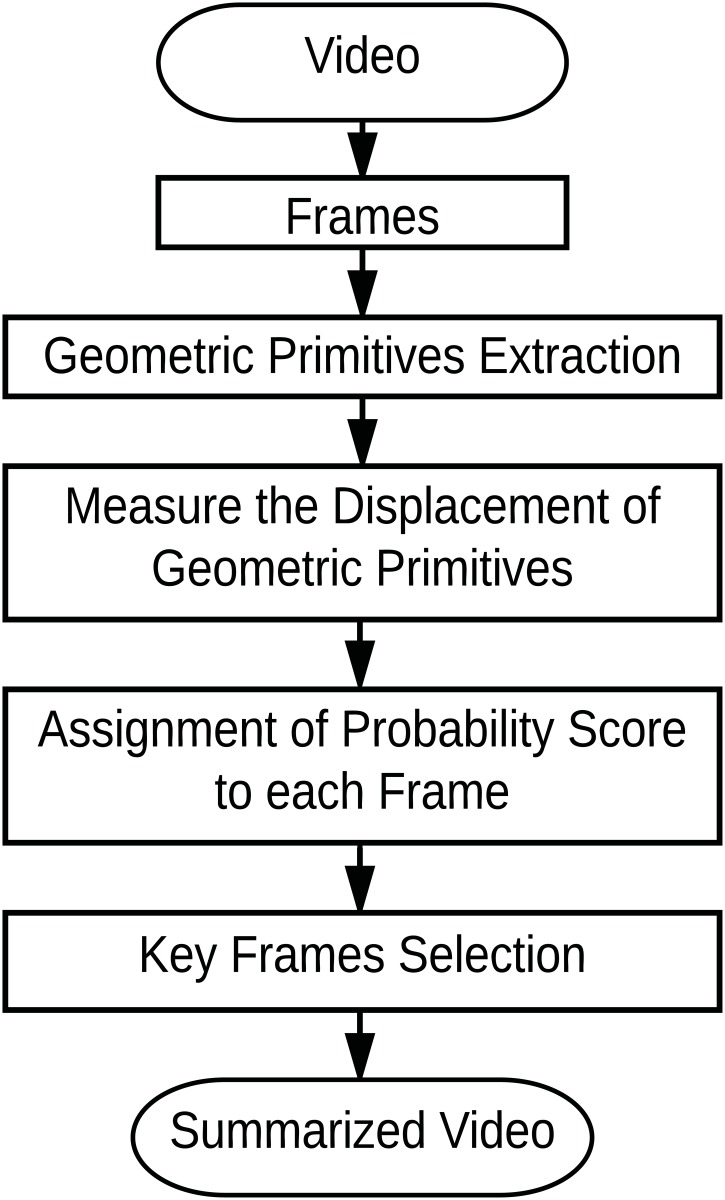
The framework of the proposed method.

The introduced method has four main steps. They are as follows- (i) Geometric primitives extraction, (ii) Measure the displacement of geometric primitives, (iii) Assignment of probability score, and (iv) Key frame selection. The main steps of the proposed method are shown in Fig 2. The details of each section is presented in subsequent sub-sections.

### Geometric primitives extraction

**Fig 3:**
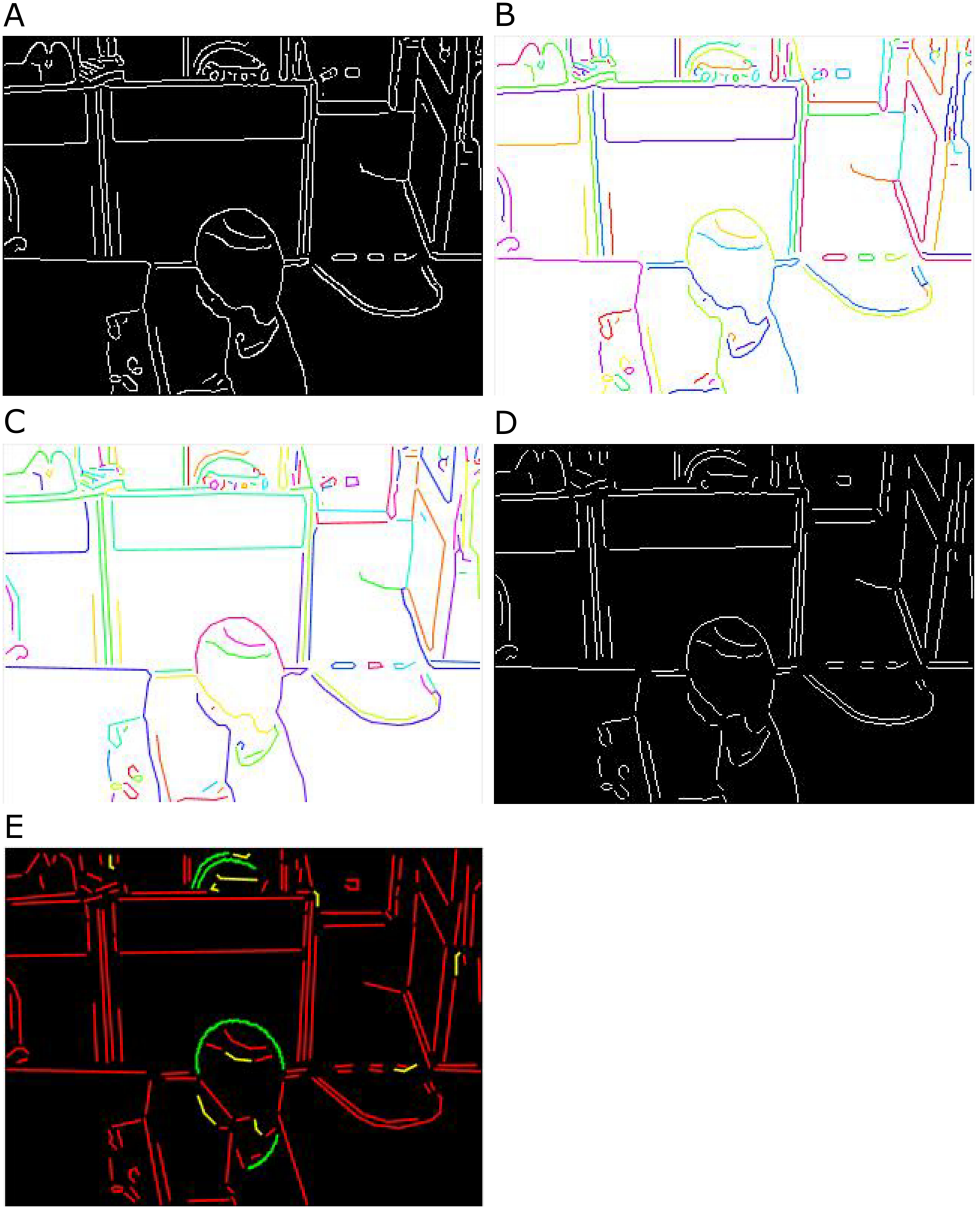
The steps to extract geometric primitives. (a) an edge map of frame number 4721 of the bl-3 video of BL-7F dataset obtained by Canny edge detector; (b) and (c) connected edge contours and corresponding line segments of (a) (different color is applied to edgelists and line segments for better visualization); (d) connected edge contour after splitting at sharp turn and inflection points; and (e) geometric primitives to represent objects (lines, angles, and conic parts are represented by red, yellow, and green).

In the proposed method, objects in a video frame are represented by geometric primitives, such as line segments, angles and conic parts. The motivation is that these primitives are independent of object size, efficient for comparisons and matching, and invariant to scale and viewpoint changes. Moreover, they are an effective feature in a challenging environment. To extract geometric primitives, the conventional Canny edge detection method is applied to obtain a binary edge image from a video frame. In Fig 3a, a binary edge image of frame (number 4721) from the bl-3 video of BL-7F dataset [1] is shown. After obtaining the binary edge image, lists of connected edge points (edgelists) without any branch and fitted straight line segments to connected edge points are obtained by applying the method developed by Kovesi [12]. Each line segment may contain single or multiple lines. The connected edge points and the corresponding fitted line segments are shown in Figs 3b and 3c respectively obtained from the binary edge image of Fig 3a. Different color is applied to edgelists and line segments for better visualization. Later, sharp turn and inflection points are identified and line segments are split at these points as per the method proposed in [46]. The connected edge contours after splitting at sharp turn and inflection points are shown in Fig 3d.

A single line segment is considered as a straight line (Φ) (red lines in Fig 3e). Line segments with two lines are modeled as corners (Ω) (yellow lines in Fig 3e). The connected edge segments whose corresponding line segments have more than two lines are matched with conic (circle, ellipse, parabola, and hyperbola) parts using Pascal’s theorem [13] [14].

**Fig 4:**
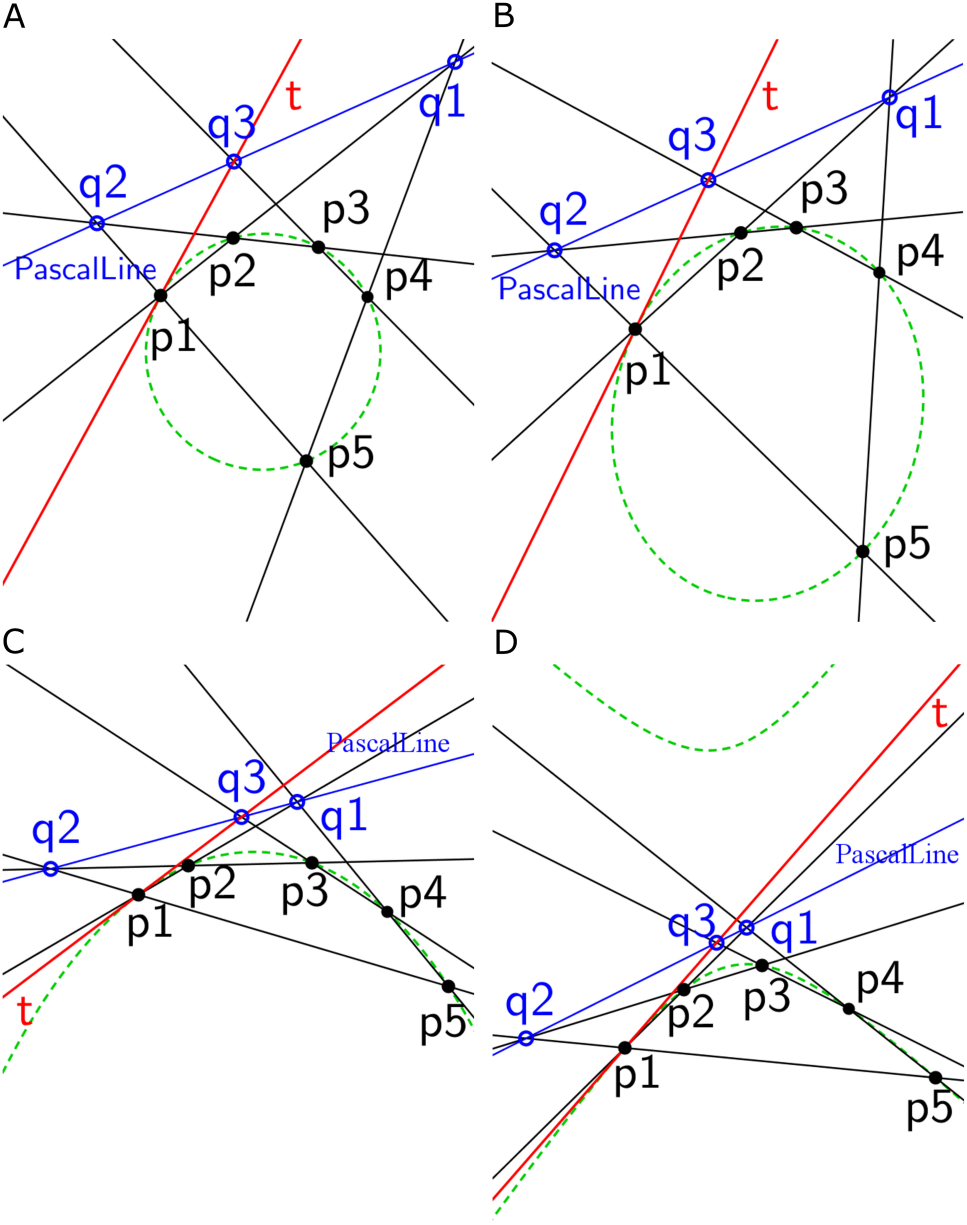
The construction of tangent (red line) on a point of the conic sections (green dotted curve) using Pascal’s theorem; *p*1, *p*2, *p*3, *p*4, and *p*5 are five points on the conic sections. Tangents (*t*) are drawn on the point *p*1 of (a) a circle, (b) an ellipse, (c) a parabola and (d) a hyperbola. The Pascal line (blue line) is represented by *q*1*q*2*q*3.

To validate an edge segment as a conic part, tangents are drawn first at each endpoint of an edge segment and an arbitrary point on the edge segment is required. The existing methods for tangent estimation construct a tangent on a digital curve based on a parameter [18] [19]. However, finding an optimal parameter value is one of the main problems for these methods. Therefore, a new parameterless tangents estimation method is proposed based on Pascal’s theorem [13] [14]. Consider *p*1, *p*2, *p*3, *p*4 and *p*5 are five points of a circle (Fig 4a), an ellipse (Fig 4b), a parabola (Fig 4c) and a hyperbola (Fig 4d). To avoid an exceptional case of Pascal’s theorem, these five points are selected in such a way that no three co-linear and no parallel lines can be formed using these points. The point *q*1 is the intersection of line *p*1*p*2 and line *p*5*p*4, and *q*2 is the intersection of *p*5*p*1 and *p*3*p*2 respectively. Accordingly, *p*4*p*3 and *q*1*q*2 must meet at point *q*3. The line (*q*1*q*2*q*3) is Pascal line and is represented by a blue line. The expected tangent line (*t*) of the conic at point *p*1 is obtained by connecting *p*1 and *q*3. Similarly, we can get a tangent line at any point of the conics. In this way, two tangents (*t*1 and *t*2) are constructed at each end point (*p*1 and *p*5) of the edge segment.

These tangents are then used to construct a conic part using Pascal’s theorem. In our method, five points are obtained from an edge segment so that these points can divide it into four equal parts. We follow this approach as it represents the conic more accurately than random sampling.

**Algorithm 1** getTangent(*p*1, *p*2, *p*3, *p*4, *p*5)

Begin

 Find the intersecting point *q*1 between *p*1*p*2 and *p*5*p*4

 Find the intersecting point *q*2 between *p*5*p*1 and *p*3*p*2

 Find the intersecting point *q*3 between *p*4*p*3 and *q*1*q*2

 Draw the tangent *t* on *p*1 by connecting *p*1 and *q*3

End

In the real world, part of an object can be a circular or an elliptical or a parabolic or a hyperbolic curve. As a result, the object detection methods proposed with the circular arc does not fit accurately with an elliptical or a parabolic or a hyperbolic curve and vice versa. Therefore, an innovative conic part construction method is introduced.

**Fig 5:**
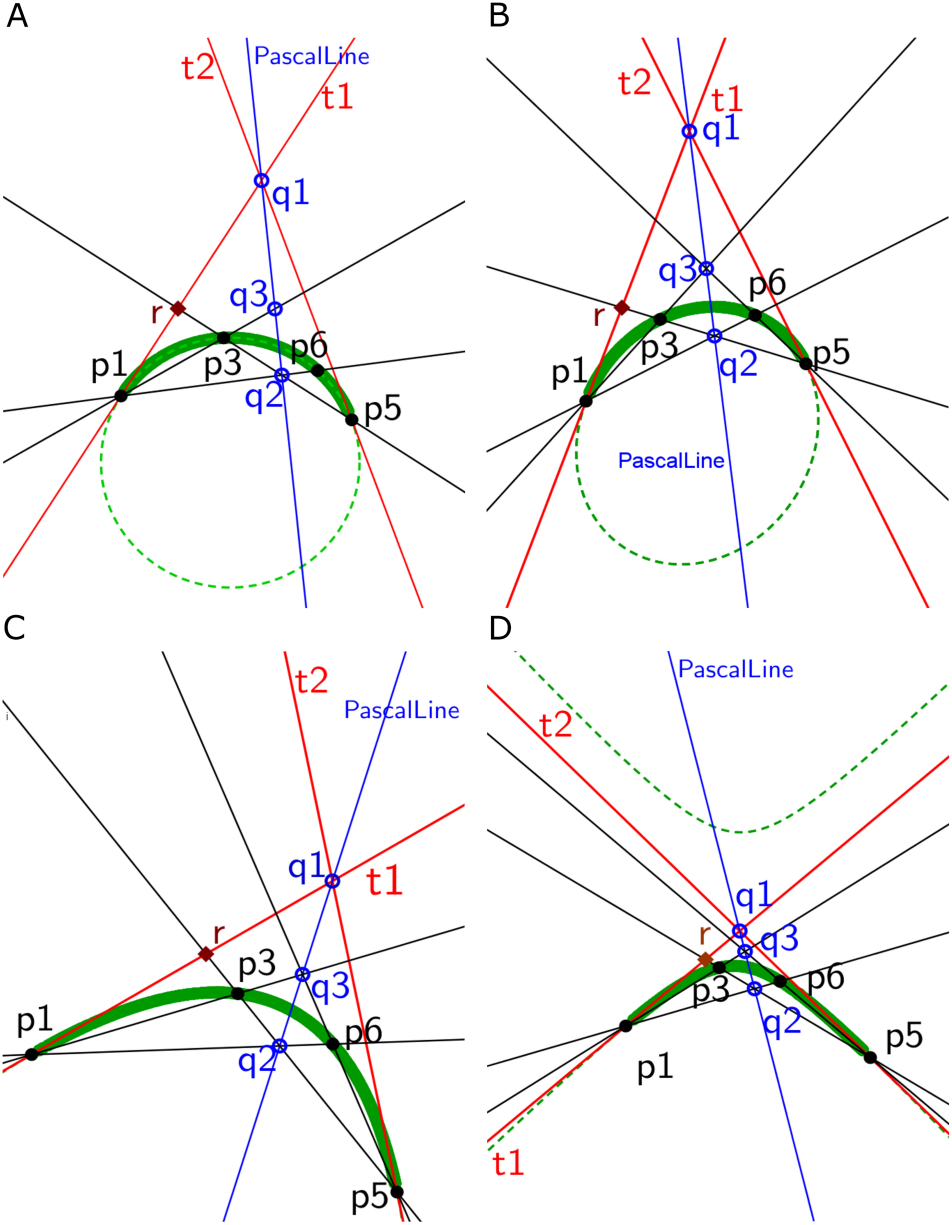
Conic part construction using Pascal’s theorem. Two tangents *t*1 and *t*2 (red lines) are constructed on start point *p*1 and end point *p*5 respectively and a point (*p*3) is taken from (a) a circle, (b) an ellipse, (c) a parabola and (d) a hyperbola. the line *q*1*q*2*q*3 is Pascal line (blue line). The point *p*6 is another point on conic sections obtained by Pascal’s theorem. If the point *q*2 moves from point *r* to *p*5 along the *rp*5 line, the conic part *p*1*p*3*p*5 (solid green curve) is constructed. The conic sections are represented by dotted green color.

Using two tangents (*t*1 and *t*2) and a selected point (*p*3) from the edge segment, a conic part is constructed based on Pascal’s theorem. Consider, tangents *t*1 and *t*2 at *p*1 (start point) and *p*5 (end point) of a circle (Fig 5a), an ellipse (Fig 5b), a parabola (Fig 5c) and a hyperbola (Fig 5d). These tangents (*t*1 and *t*2) intersect at point *q*1. The tangent *t*1 and *p*5*p*3 meet at point *r*. The point *q*2 is selected from line *p*5*r*. The intersecting point (*q*3) is obtained from *q*1*q*2 and *p*1*p*3. The line *q*1*q*2*q*3 is Pascal line (blue line in Fig 5). Finally, a point (*p*6) on the conic is obtained by intersecting *p*1*q*2 and *q*3*p*5. If the point *q*2 is moved from *r* to *p*5, the conic part *p*1*p*3*p*5 is obtained. Following this process, a conic part is obtained for the corresponding edge segment. The edge segments are fitted with the corresponding conic parts using the Least Square Fitting (LSF) method with residual two pixels.

**Algorithm 2** getConicPart(*p*1, *p*3, *p*5, *t*1, *t*2)

Begin

 Find the intersecting point *q*1 between *t*1 and *t*2

 Find the intersecting point *r* between *t*1 and *p*5*p*3

 Select a point *q*2 from *p*5*r*

 Find the intersecting point *q*3 between *q*1*q*2 and *p*1*p*3

 Find the intersecting point *p*6 between *p*1*q*2 and *q*3*p*5

 Move *q*2 from *r* to *p*5, *p*6 will move from *p*1 to *p*5 and conic part *p*1*p*3*p*5 will be constructed connecting *p*1, *p*3 and *q*5

End

If the connected edge segments fit with conic parts obtained by Pascal’s theorem, these conic parts represent curve segments (Ψ) of objects in a video frame (see green curve in Fig 3e). Otherwise, connected edge segments are represented by the corresponding line segments. The points of Φ, Ω and Ψ are provided with a value of one, two, and three respectively to distinguish them separately.

### Measure the displacement of geometric primitives

Object activities are indicators of events within a video [2]. Furthermore, human beings pay more attention to dynamic objects than those that are static [4]. Therefore, a new approach is proposed to measure the activities of objects.

To obtain the activities of objects pixel wise comparison is performed between geometric primitives of the current frame and the previous frame. Suppose, current frame (*F*_*n*_) and previous frame (*F*_(*n* − 1)_) with geometric primitives are denoted by *G*_*n*_ and *G*_(*n* − 1)_ respectively where n = 2, 3, …., N (total number of frames in a video). The pixel values of *G*_*n*_ or *G*_(*n* − 1)_ are between zero and three where zero, one, two and three represent background, Φ, Ω and Ψ respectively. The pixel locations of each Φ, Ω and Ψ of the current frame are compared with those of the previous frame. Consider a line segment $\Phi_{n}^{i}$, (where i = 1, 2, 3, …., I total number of line segments in *G*_*n*_ and *n* represents that it belongs to *n*th number of frame with geometric primitives, *G*_*n*_) contains A×2 array of (row $\Phi_{n}^{i}$ (a, 1), column $\Phi_{n}^{i}$(a, 2)) coordinates of pixels where *a* = 1, 2, 3, …, *A* total number of pixels in $\Phi_{n}^{i}$. The value of *G*_*n*_ ($\Phi_{n}^{i}$ (a, 1),$\Phi_{n}^{i}$ (a, 2)) is one as the pixel value of the line segment is set to one. If a pixel location ($\Phi_{n}^{i}$ (a, 1),$\Phi_{n}^{i}$ (a, 2)) of $\Phi_{n}^{i}$ from *G*_*n*_ is also a pixel of a straight line from the previous frame with geometric primitives *G*_(*n* − 1)_, the pixel ($\Phi_{n}^{i}$ (a, 1),$\Phi_{n}^{i}$ (a, 2)) is considered as a stationary pixel. Otherwise, it is considered as a dynamic pixel. To obtain this information, the pixel value at $\Phi_{n}^{i}$ (a, 1),$\Phi_{n}^{i}$ (a, 2) in the previous frame with the geometric primitives *G*_(*n* − 1)_ is calculated. If the value of *G*_(*n* − 1)_ ($\Phi_{n}^{i}$ (a, 1),$\Phi_{n}^{i}$ (a, 2)) is also one (as pixel value one denotes a straight line), the pixel ($\Phi_{n}^{i}$ (a, 1),$\Phi_{n}^{i}$ (a, 2)) is regarded as a similar pixel and is assigned value zero. Otherwise, it is consider as a dissimilar pixel and assigned value one. Therefore, the positional dissimilarity *D* of $\Phi_{n}^{i}$ in *G*_*n*_ with respect to *G*_(*n* − 1)_ is calculated by the following equation:-

$$\begin{array}{c} D\left(a\right)=\{\begin{array}{cc} 1 & \text{if}\;G_{n}\left(\Phi_{n}^{i}\left(a,1\right),\Phi_{n}^{i}\left(a,2\right)\right)\neq G_{\left(n-1\right)}\left(\Phi_{n}^{i}\left(a,1\right),\Phi_{n}^{i}\left(a,2\right)\right),\\ 0 & \text{otherwise} \end{array} \end{array}$$ (1)

where *a* = 1, 2, 3, …, *A* total number of pixels in $\Phi_{n}^{i}$, and *D* is A×1 array as it contains either 0 or 1.

**Fig 6:**
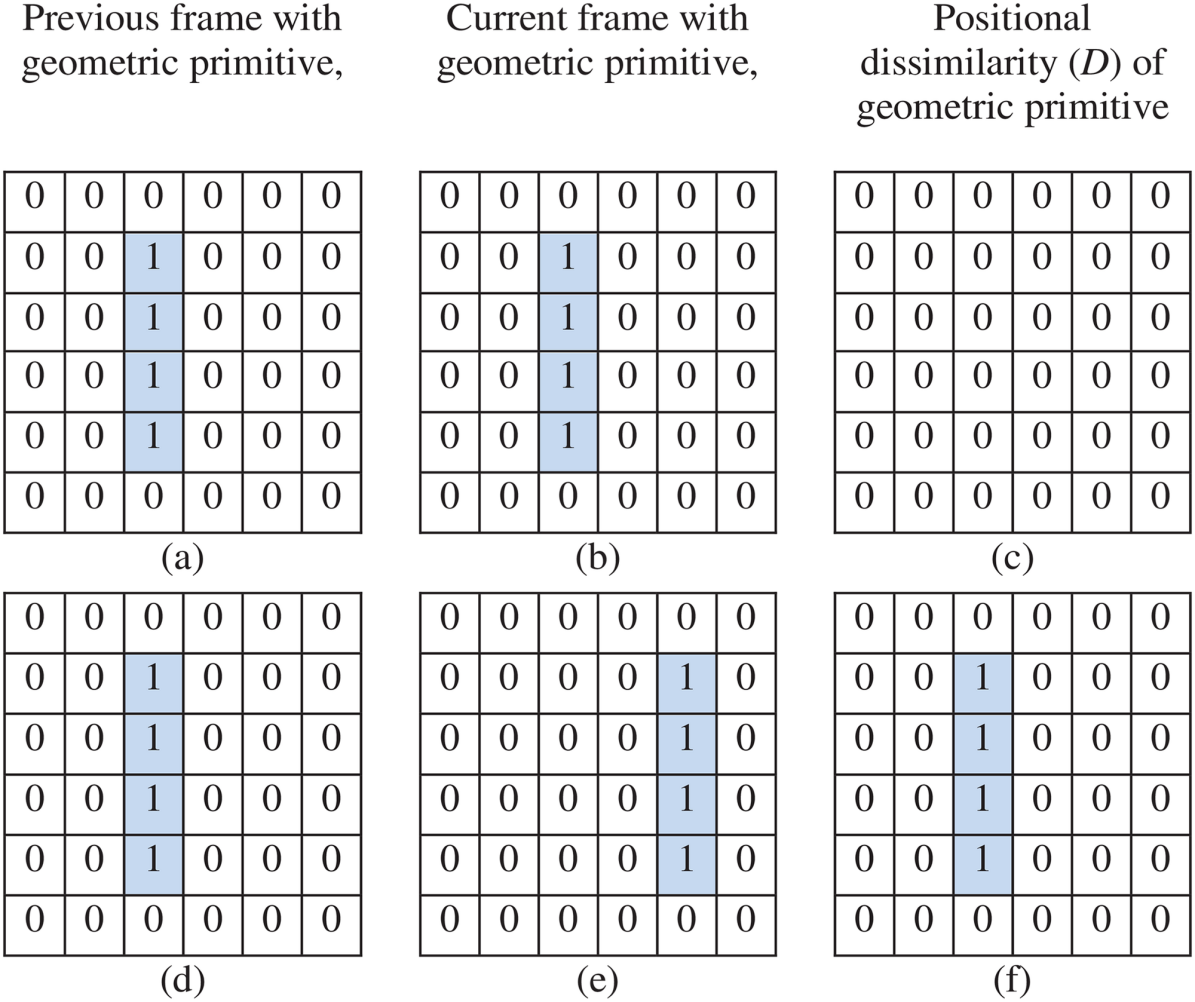
The explanation of the method to measure the dissimilarity of geometric primitives. (a) and (b) are the previous and the current frame with a straight line respectively, (c) is the dissimilar points of a straight line in (b) with respect to (a). Similarly, (d) and (e) are the previous and the current frame with a straight line respectively; (f) is the dissimilar points of a straight line in (e) with respect to (d).

In Fig 6, a straight line segment $\Phi_{n}^{i}$ (i = 1) (light blue color) and background (white color) are represented by pixel value one and zero respectively. The total number of pixels in $\Phi_{n}^{1}$ is four (a = 1, 2, 3, 4 (A = 4)). In Fig 6b, the pixel coordinates of $\Phi_{n}^{1}$ in the current frame with geometric primitives *G*_*n*_ are ($\Phi_{n}^{1}$ (1, 1), $\Phi_{n}^{1}$ (1, 2)), ($\Phi_{n}^{1}$ (2, 1), $\Phi_{n}^{1}$ (2, 2)), ($\Phi_{n}^{1}$ (3, 1), $\Phi_{n}^{1}$ (3, 2)), ($\Phi_{n}^{1}$ (4, 1), $\Phi_{n}^{1}$ (4, 2)) = ((2, 3), (3, 3), (4, 3), (5, 3)) respectively. The pixel values of these coordinates in *G*_*n*_ are *G*_*n*_(2, 3), *G*_*n*_(3, 3), *G*_*n*_(4, 3), and *G*_*n*_(5, 3) = 1, 1, 1, and 1 (Fig 6b). The pixel values of these coordinates in *G*_*n* − 1_ are *G*_*n* − 1_(2, 3), *G*_*n* − 1_(3, 3), *G*_*n* − 1_(4, 3), and *G*_*n* − 1_(5, 3) = 1, 1, 1, and 1 (Fig 6a). Therefore, the dissimilarity D(1), D(2), D(3), and D(4) = 0, 0, 0, and 0 (Fig 6c). Similarly in Fig 6e, the pixel coordinates of $\Phi_{n}^{1}$ in the current frame with geometric primitives *G*_*n*_ are ($\Phi_{n}^{1}$ (1, 1), $\Phi_{n}^{1}$ (1, 2)), ($\Phi_{n}^{1}$ (2, 1), $\Phi_{n}^{1}$ (2, 2)), ($\Phi_{n}^{1}$ (3, 1), $\Phi_{n}^{1}$ (3, 2)), ($\Phi_{n}^{1}$ (4, 1), $\Phi_{n}^{1}$ (4, 2)) = ((2, 5), (3, 5), (4, 5), (5, 5)) respectively. The pixel values of these coordinates in *G*_*n*_ are *G*_*n*_(2, 5), *G*_*n*_(3, 5), *G*_*n*_(4, 5), and *G*_*n*_(5, 5) = 1, 1, 1, and 1 (Fig 6e). The pixel values of these coordinates in *G*_*n* − 1_ are *G*_*n* − 1_(2, 5), *G*_*n* − 1_(3, 5), *G*_*n* − 1_(4, 5), and *G*_*n* − 1_(5, 5) = 0, 0, 0, and 0 (Fig 6d). Therefore, the dissimilarity D(1), D(2), D(3), and D(4) = 1, 1, 1, and 1 (Fig 6f).

The dissimilarity score *E* of $\Phi_{n}^{i}$ in *G*_*n*_ is measured as follows:-

$$\begin{array}{c} E=\frac{\sum_{a=1}^{A}D\left(a\right)}{A} \end{array}$$ (2)

If the dissimilarity score *E* is greater than a threshold *τ*, $\Phi_{n}^{i}$ is considered as a part of a dynamic object. Otherwise, $\Phi_{n}^{i}$ is selected as part of a stationary object and it is neglected. Similarly, the dissimilarity score *E* for all geometric primitives (Φ, Ω and Ψ) in *G*_*n*_ with respect to *G*_(*n* − 1)_ is measured and categorized into a part of stationary or dynamic objects based on the threshold *τ*. The line segments (Φ), angles (Ω) and conic parts (Ψ) in *G*_*n*_ that belong to dynamic objects are denoted by *d*Φ_*n*_, *d*Ω_*n*_, and *d*Ψ_*n*_ respectively where *d* represents dynamic objects. The stationary geometric primitives are neglected.

**Fig 7:**
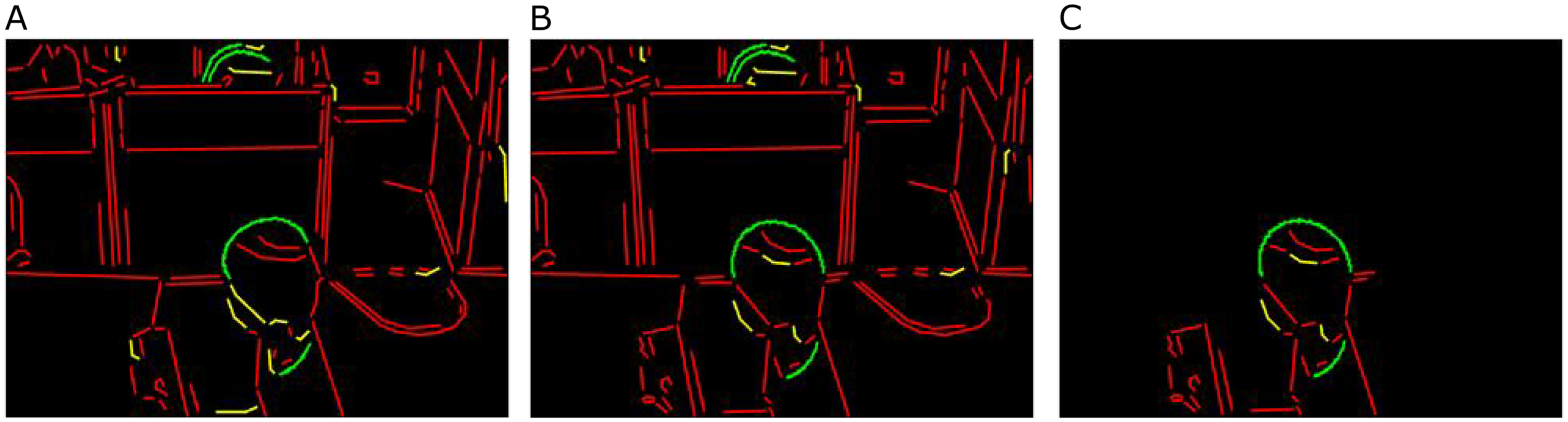
A method to calculate the displacement of geometric primitives. (a) and (b) are geometric primitives of frame number 4720 (previous frame) and 4721 (current frame) of the bl-3 video of BL-7F dataset respectively, (c) dissimilar geometric primitives of (b) with respect to (a).

The geometric primitives of frame number 4720 and 4721 from the bl-3 video of BL-7F dataset [1] are shown in Fig 7a and 7b. The dissimilar geometric primitives of frame number 4721 with respect to frame number 4720 are shown in Fig 7c.

### Assignment of probability score

In the proposed method, each frame is assigned a probability score to become a key frame. The total lengths of *d*Φ_*n*_, *d*Ω_*n*_, and *d*Ψ_*n*_ of *G*_*n*_ obtained by the previous step are measured. The probability score *W*_*n*_ of the current frame *F*_*n*_ is assigned by the following equation:-

$$\begin{array}{c} W_{n}=\sum d\Phi_{n}+\sum d\Omega_{n}+\sum d\Psi_{n} \end{array}$$ (3)

**Fig 8:**
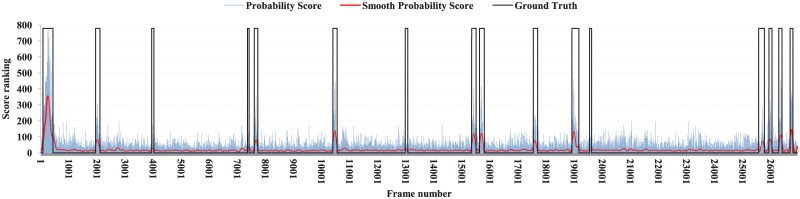
The probability score generation using geometric primitives of office-1 video. The light blue, red, and black lines represent probability score, smooth probability score by Savitzky-Golay filtering [47] and ground truth respectively.

The probability scores (*W*) for all video frames of a video are smoothed by applying Savitzky-Golay filtering [47] with window size *ω*. The main advantage of this filtering is that it enhances local maxima [47]. In Fig 8, the probability scores (*W*), smooth probability scores (*SW*) and ground truth key frames of the office-1 video are shown by light blue, and red, and black color respectively. Ground truth key frames are multiplied by maximum of *W* for better visualization.

### Keyframe selection and summary generation

In the final step, SW is sorted in ascending order so that the frame with the highest dynamic objects appears on the top of the list and frames with less or no dynamic objects remain at the bottom. As a result, a list of sorted smooth probability scores (SSW) is obtained.

The introduced approach generates summarized video based on the skimming ratio (*λ*). The proposed method enables the user to select for the value of *λ*. Otherwise, this approach selects a default value of *λ*. After that, this method selects video frames from top of the list of SSW based on *λ*. From these selected frames, frames with no dynamic object are removed. Finally, summarized video is produced from these video frames, keeping their sequential order in the original video.

## Results and discussion

The proposed method is evaluated by the publicly available BL-7F dataset [1], Office [48] and Office Lobby dataset [48]. They are considered to be the benchmark datasets to evaluate the performance of the video summarization techniques. In the BL-7F dataset, 19 surveillance videos are taken from fixed surveillance cameras located in the seventh floor of the BarryLam Building in the National Taiwan University. The duration of each video is 7 minutes 10 seconds and contains 12,900 frames. This dataset also provides a complete list of selected key frames as a ground truth for each video. In Office dataset [48], four videos are collected from stably held with non-fixed cameras. The main difficulties are the vibration of camera and different lighting conditions. Similarly, three videos are collected in Office Lobby dataset [48], with stably held but non-fixed cameras. However, they contain more crowded scenes with richer activities compared to the Lobby and Office datasets. The ground truth key frames for both the Office and Office Lobby datasets are also publicly available. No ethics approval is required for this work as no human subject is involved in any step of this work.

**Fig 9:**
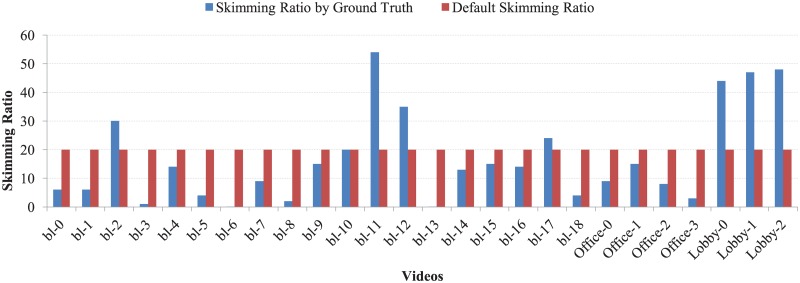
The comparison of the skimming ratio of the ground truth key frames with respect to the total video frames and default skimming ratio (20% of total video frames) for BL-7F, lobby, and office datasets.

In this experiment, the value of the dissimilarity threshold *τ* was set to 0.85. Experimental results revealed that this value satisfied the condition to identify dynamic geometric primitives successfully. The window size *ω* for Savitzky-Golay filtering [47] was set to 300. This value effectively highlighted the key frames and suppressed the unnecessary frames (see Fig 8). We apply Canny edge detector with the default parameters provided in Matlab (https://au.mathworks.com/products/matlab.html) similar to [49] to extract edges from all video frames. Matlab selects the high and low value of the sensitivity threshold to the highest value of the gradient magnitude of the image and 0.4×the high value respectively. Matlab also selects the default value of the standard deviation of the Gaussian filter to $\sqrt{2}$. black The skimming ratio *λ* (user preferred) was set to the skimming ratio of the ground truth key-frames for each video in the datasets [1] [48] plus ten per cent of *λ*. This value ensured more accurate summarization results. The default value of *λ* is set to 20% of the total number of frames of a video. This skimming ratio is also consistent with some other existing methods [50] [51]. In Fig 9, the skimming ratio of the ground truth key frames and the total number of frames and the default skimming ratio (20% of the total video frames) for BL-7F, Lobby, and Office are shown. It is clear from the graph that the default skimming ratio is almost consistent with the ground truth skimming ratio provided in [1] [48].

An objective evaluation was performed to justify the effectiveness of the proposed method. In this regards, a set of objective evaluation metrics, such as precision, recall and F1-measure were computed. The definition of precision and recall are as follows

$$\begin{array}{c} Precision=\frac{t_{p}}{t_{p}+f_{p}} \end{array}$$ (4)

$$\begin{array}{c} Recall=\frac{t_{p}}{t_{p}+f_{n}} \end{array}$$ (5)

where *t*_*p*_ is the number of key-frames selected by the proposed method, *f*_*p*_ is the number of frames that are not key-frame selected by the proposed method, and *f*_*n*_ is the number of key-frames not selected by the proposed method. However, they alone cannot provide an unbiased measurement of the performance of the proposed method. For example, a method with high precision and poor recall or vice versa cannot be an excellent method. Therefore, a method with both higher precision and recall is an excellent approach. To represent this measure, F1-measure is defined as combining both precision and recall and is represented as follows:-

$$\begin{array}{c} F_{1}-measure=2\times \frac{Precision\times Recall}{Precision+Recall} \end{array}$$ (6)

**Fig 10:**
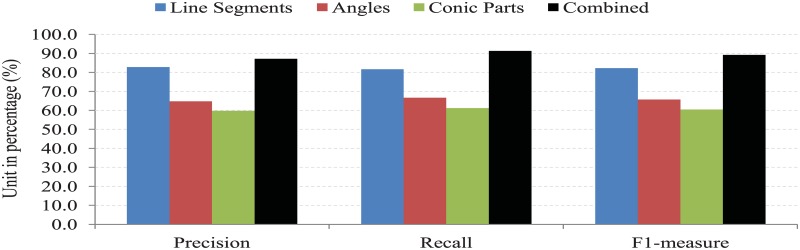
The comparative analysis of line segments, angles, conic parts and the combination of them for summarizing bl-0 video of BL-7F dataset.

The amount of conic parts in a video frame is very low compared to other geometric primitives, such as line segments and angles (corners). Among all the conic parts, elliptical segments may exist more than circular, parabolic or hyperbolic segments. However, conic parts still have a significant role to detect dynamic objects and to summarize a video. To identify the role of each geometric feature, such as line segments, angles, and conic parts for detecting dynamic objects and generating the summary of a video, we compare the result obtained by the individual geometric primitive (feature) on bl-0 video of BL-7F dataset. In Fig 10, the precision, recall and F1-measure of the proposed method with employing only Φ, Ω, Ψ and combination of them in bl-0 video of BL-7F dataset are displayed. From this figure, it is easily observed that the proposed method with only Φ shows better than that of applying only Ω or Ψ. The proposed method with only Ψ performs less compared to the proposed method with only Φ or Ω. However, the combination of Φ, Ω, Ψ outperforms the individual features. Therefore, we combine Φ, Ω, Ψ in the proposed method for video summarization.

**Fig 11:**
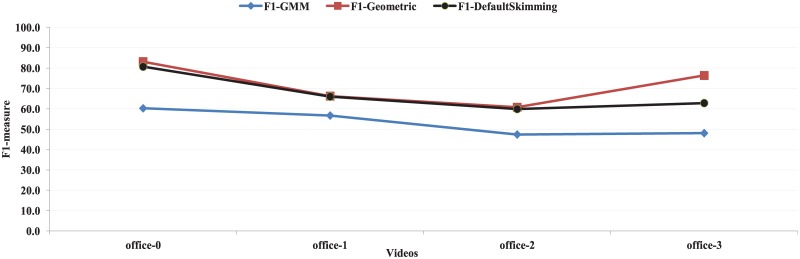
The graphical representation of F1-measure of the proposed with user provided skimming ratio (F1-Geometric) and default skimming ratio (F1-DefaultSkimming) and GMM based approach (intra-view) (F1-GMM) on office dataset.

The proposed approach is compared with the single-view summarization of the GMM based method [1] as the proposed method is designed for the single-view summarization. This method is recently proposed and the state of the art method to surveillance videos as it outperforms relevant and recent methods [1]. As the proposed method is implemented on surveillance video, GMM based method is selected to compare with. The precisions, recalls and F1-measures of the proposed method and GMM-based method on Office dataset are shown in Table 1. The values of F1-measure of the proposed method for office-0, office-1, office-2, and office-3 videos are 83.2, 66.2, 60.7, and 76.4 respectively. On the other hand, the GMM based method (intra-view) shows 60.2, 56.7, 47.4 and 48.0 respectively. From this table, it is clear that the F1-measures of the proposed method are higher than those of the GMM based method for all four videos. The graphical representation of the F1-values of the proposed method with user provided skimming ratio (F1-Geometric) and default skimming ratio (F1-DefaultSkimming) and GMM based method (F1-GMM) is shown in Fig 11.

**Table 1:** Precision, recall, and F1-measure of GMM (intra-view) and the proposed method for office dataset.

Video	GMM (intra-view)			The Proposed Method		
	Precision (%)	Recall (%)	F1-measure (%)	Precision (%)	Recall (%)	F1-measure (%)
office-0	47.0	83.9	60.2	83.2	83.2	83.2
office-1	42.7	84.3	56.7	66.2	66.2	66.2
office-2	31.2	98.5	47.4	52.1	72.9	60.7
office-3	34.2	80.7	48.0	76.4	76.3	76.4

**Fig 12:**
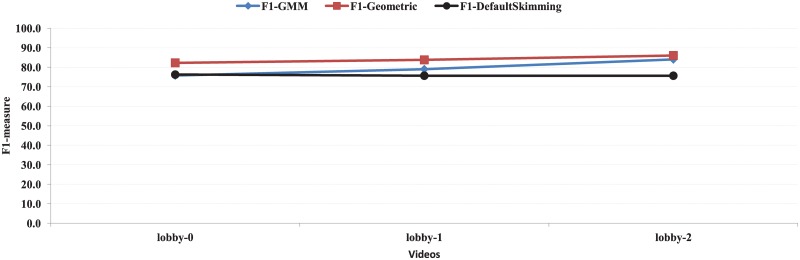
F1-measure of the proposed with user provided skimming ratio (F1-Geometric) and default skimming ratio (F1-DefaultSkimming) and GMM based approach (intra-view) (F1-GMM) for lobby dataset.

In Table 2, the results of the precision, recall, and F1-measure for Lobby dataset [48] obtained by both the GMM-based method (intra-view) and the proposed method are presented. The values of F1-measure obtained by the proposed method for lobby-0, lobby-1, and lobby-2 are 81.6, 83.8, and 86.0 respectively. In comparison, the GMM based method (intra-view) obtains 75.8, 79.0, and 84.0 respectively. Therefore, the proposed method outperforms the GMM based method for the Lobby dataset. In Fig 12, the results of F1-measure for four videos of the Lobby dataset [48] obtained the GMM based method (intra-view) (F1-GMM) and the proposed method with user provided skimming ratio (F1-Geometric) and default skimming ratio (F1-DefaultSkimming) are shown.

**Table 2:** Precision, recall, and F1-measure of GMM (intra-view) and the proposed method for lobby dataset.

Video	GMM (intra-view)			The Proposed Method		
	Precision (%)	Recall (%)	F1-measure (%)	Precision (%)	Recall (%)	F1-measure (%)
lobby-0	65.3	90.2	75.8	73.2	93.9	82.3
lobby-1	70.0	91.8	79.0	75.2	94.6	83.8
lobby-2	76.0	93.2	84.0	81.4	95.7	88.0

**Fig 13:**
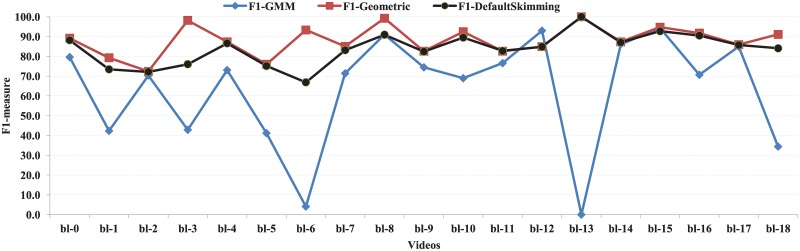
F1-measure of the proposed with user provided skimming ratio (F1-Geometric) and default skimming ratio (F1-DefaultSkimming) and GMM based approach (intra-view) (F1-GMM) for Bl-7F dataset.

The results of the precision, recall, and F1-measure obtained by both the GMM-based method (intra-view) and the proposed method for the BL-7F dataset are provided in Table 3. The values of F1-measure obtained by the proposed method are higher for 18 videos out of 19 videos than those of the GMM based method. In Fig 13, the results of F-1 measure obtained the GMM based method (F1-GMM) and the proposed method with user provided skimming ratio (F1-Geometric) and default skimming ratio (F1-DefaultSkimming) for 19 videos of BL-7F dataset are represented. From this graph, it is apparent that the proposed method obtains slightly better results than the GMM based method for four videos, such as bl-2, bl-14, bl-15, and bl-17 videos. Noticeable enhanced values of F1-measure are obtained by the proposed method for six videos namely bl-0, bl-4, bl-7, bl-8, bl-9 and bl-11. The proposed method achieves superior performance for the remaining nine videos.

**Table 3:** Precision, recall, and F1-measure of GMM (intra-view) and the proposed method for Bl-7F dataset.

Video	GMM (intra-view)			The Proposed Method		
	Precision (%)	Recall (%)	F1-measure (%)	Precision (%)	Recall (%)	F1-measure (%)
bl-0	70.3	91.8	79.6	87.1	91.4	89.2
bl-1	28.9	79.0	42.3	74.2	85.2	79.3
bl-2	77.8	64.2	70.3	70.1	86.9	77.6
bl-3	27.4	98.5	42.9	98.5	97.8	98.2
bl-4	62.4	88.1	73.1	80.1	96.1	87.3
bl-5	26.1	97.6	41.2	66.2	89.2	76.0
bl-6	2.1	100.0	4.1	87.5	100.0	93.3
bl-7	63.9	80.9	71.4	85.1	85.0	85.0
bl-8	83.6	100.0	91.1	99.5	99.0	99.2
bl-9	97.5	60.2	74.4	82.6	82.6	82.6
bl-10	61.4	78.6	68.9	86.2	99.4	92.3
bl-11	98.3	62.7	76.6	82.6	82.6	82.6
bl-12	88.3	98.2	93.0	79.4	91.3	84.9
bl-13	0.0	0.0	0.0	100.0	100.0	100.0
bl-14	76.9	97.7	86.1	85.3	95.6	90.2
bl-15	92.4	96.5	94.4	95.3	98.2	96.7
bl-16	55.6	97.0	70.7	91.8	91.7	91.8
bl-17	88.1	81.9	84.9	91.6	87.4	89.5
bl-18	21.3	89.2	34.4	91.2	91.0	91.1

The reasons for the failure of the proposed method to perform better in bl-12 video of BL-7F dataset have been evaluated. In bl-12 video, some ground truth key-frames do not contain any dynamic objects or object activities. Similarly, some frames are not selected as ground truth key-frames although they contain significant dynamic object or object activities as provided in [1]. For example, frame no 4083, 4120, and 4563 show a person is working near the door. However, these frames are not selected as ground truth key frames. On the other hand, frame no 12615, 12675, and 12750 do not contain any object activities. However, they are selected as ground truth key-frames. There is no explanation found for this incident in [1].

After observing the proposed method both quantitatively and qualitatively, it is certain that the proposed method based on geometric primitives performs better than the GMM based method (intra-view) [1]. The main reason for this success is that the proposed method utilizes geometric primitives, such as line segments, angles, and conic parts for object detection, and applies a dissimilarity measure method to include the degree of object activities. In contrast, the GMM based method ranks the video frames based on the size of the foreground objects in the intra-view stage [1]. This method does not consider multi-modal phenomena such as illumination change, variation of local motion, or occlusion. Therefore, the proposed method performs better than the GMM based method.

## Conclusion

In this paper, an innovative approach is proposed to summarize video using geometric primitives, such as line segments, angles, and conic parts. Existing video summarization methods fail to detect dynamic objects in low contrast regions. However, edges are prominent in low contrast regions. Again, to represent objects, geometric primitives (such as lines, arcs) are higher level and more distinguishable descriptors than edges. Existing object detection methods apply circular or elliptical arcs or entire circles or ellipses for object segment representation. However, elliptical arcs do not fit accurately to circular curves or vice-versa. Therefore, a conic part is applied for fitting the curve segments. To measure the activities of objects, a new cost function is proposed to calculate the displacements of geometric primitives between two successive frames. Experimental results has shown that the proposed summarization method using geometric primitives outperforms the recent state-of-the-art method. The proposed method performs very well in case of stationary camera. In the future, we will consider the moving camera for video summarization.
